# Supplementary material for: Sterilization Effects on Liposomes with Varying Lipid Chains
Source: Nanomaterials (Basel). 2025 Sep 27;15(19):1478. doi: 10.3390/nano15191478 (PMC12526366; doi:10.3390/nano15191478)
Supplement: Supplementary file 1 [file nanomaterials-15-01478-s001.zip › nanomaterials-3856912-supplementary.pdf]

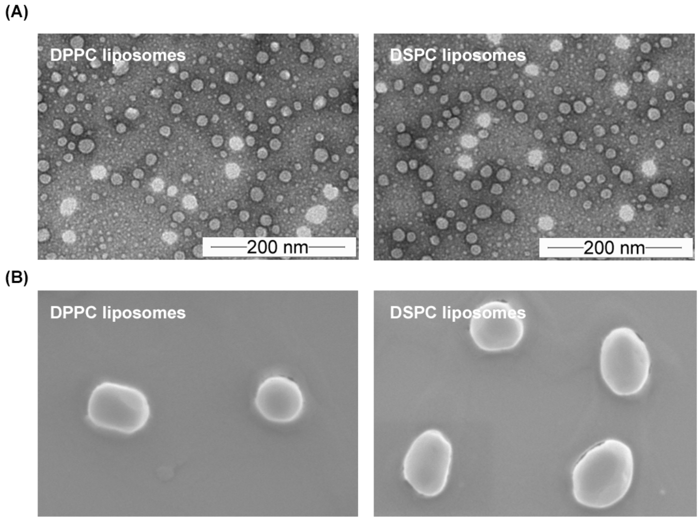

Figure S1. Morphological characterization of DPPC and DSPC liposomes after filtration. (A) Transmission electron microscopy (TEM) images of DPPC and DSPC liposomes. (B) Scanning electron microscopy (SEM) images of the same liposomal formulations following filtration sterilization.

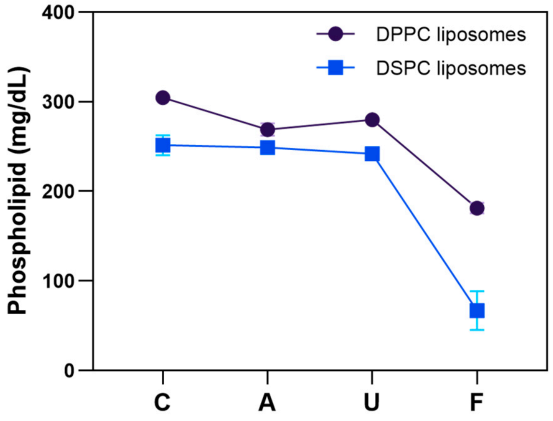

Figure S2. Phospholipid content in DPPC and DSPC liposomes before and after sterilization. Comparison of phospholipid retention in DPPC and DSPC liposomes following autoclaving, UV irradiation, and filtration.

Table S1. Percentage of cell viability of DPPC and DSPC liposomes before and after sterilization (autoclaving, UV irradiation, and filtration) at different concentrations. Data are presented as mean ± SD (n = 3).

DPPC liposomes	HaCaT								
	Doses (mg/mL)	Control		Autoclaving		UV		Filtration	
		% Cell viability		% Cell viability		% Cell viability		% Cell viability	
		24 h	48 h	24 h	48 h	24 h	48 h	24 h	48 h
	0.2	7.11±0.31	4.92±0.13	6.85±0.33	4.83±0.06	7.14±0.29	4.91±0.19	28.76±6.60	16.50±5.87
	0.1	30.07±2.08	5.18±0.22	15.53±1.36	5.00±0.16	23.83±0.79	5.15±0.38	42.35±2.96	32.99±11.75
	0.05	92.25±6.87	70.66±2.85	73.60±10.65	57.19±4.63	78.57±3.10	64.42±4.62	93.34±5.33	87.64±6.65
	0.03	102.14±3.55	87.47±4.61	101.65±8.98	85.48±6.51	92.56±8.52	86.02±3.60	98.52±7.36	96.16±10.01
	0.01	109.60±5.02	88.34±5.53	100.35±15.59	85.85±4.17	101.61±19.54	83.77±9.00	101.90±4.29	104.47±9.70
	HepG2								
	0.2	9.26±0.21	4.64±0.24	8.57±0.75	4.71±0.16	8.89±0.40	4.75±0.26	26.62±4.62	15.53±4.13
	0.1	35.95±4.48	12.36±0.96	28.06±3.96	7.67±1.82	37.81±1.75	9.79±1.13	51.94±8.46	31.06±8.25
	0.05	59.62±5.58	63.92±9.01	57.15±2.35	61.65±5.18	58.24±3.79	57.20±3.35	72.75±6.13	90.49±15.60
	0.03	77.74±8.15	103.98±7.49	74.61±4.62	88.85±4.40	83.74±3.56	90.86±3.69	87.11±7.86	105.94±3.99
	0.01	97.32±7.52	110.91±1.23	85.22±8.02	91.07±3.09	102.77±2.18	97.74±5.99	84.06±11.11	112.02±2.51
	HK-2								
	0.2	46.40±3.04	6.61±0.32	31.31±3.31	6.65±0.46	40.54±3.56	6.72±0.47	32.75±1.73	25.53±1.64
	0.1	103.18±10.03	91.06±5.09	90.18±1.93	74.38±2.38	89.31±6.09	80.35±1.90	67.10±1.70	53.65±2.35
	0.05	101.87±3.27	100.51±4.26	95.65±11.82	87.22±4.32	99.27±6.75	92.00±6.04	103.93±3.84	102.76±12.18
	0.03	100.20±1.11	96.47±12.04	89.96±7.78	96.83±4.98	94.93±11.43	92.66±5.87	100.74±4.67	102.93±10.50
	0.01	97.54±2.55	104.57±1.97	86.62±2.79	92.91±4.75	90.58±11.58	95.74±1.73	104.27±6.37	100.02±10.99
DSPC liposomes	HaCaT								
	0.28	7.22±0.60	5.30±0.22	6.85±0.11	5.42±0.13	7.04±0.32	5.57±0.08		
	0.14	73.83±8.91	59.37±3.32	57.82±5.18	41.66±2.19	64.18±3.97	57.45±3.16		
	0.07	100.34±2.37	102.40±4.01	82.45±3.31	77.79±1.26	91.06±5.79	88.55±4.36		
	0.04	104.17±7.88	100.08±3.12	88.02±1.59	86.84±4.30	97.59±3.14	98.06±2.41		
	0.02	104.47±7.92	106.04±6.35	97.56±2.26	96.47±5.21	99.77±2.36	100.23±3.54		
	0.48							26.64±0.99	29.86±0.93
	0.24							90.85±5.50	64.78±1.87
	0.12							99.23±1.13	84.87±3.34
	0.06							103.06±4.04	94.62±3.95
	0.03							101.68±3.82	90.76±5.23
	HepG2								
	0.28	6.94±0.65	4.85±0.18	7.25±0.22	5.05±0.19	7.02±0.99	4.88±0.25		
	0.14	42.19±2.14	30.87±1.73	31.63±1.71	27.04±8.85	38.87±4.05	26.72±1.86		
	0.07	70.70±4.22	66.79±2.54	57.20±2.71	48.32±1.37	82.14±0.34	76.33±2.38		
	0.04	94.02±4.24	74.02±2.27	88.32±1.41	77.59±5.45	100.49±2.36	92.58±0.80		
	0.02	106.24±1.44	101.14±1.72	100.71±1.92	102.46±1.70	102.68±2.55	101.14±0.99		
	0.48							16.63±3.00	18.03±2.56
	0.24							33.33±2.15	19.67±3.46
	0.12							83.47±2.00	81.57±1.11
	0.06							97.83±6.66	96.40±1.44
	0.03							97.40±4.13	107.10±1.53
	HK-2								
	0.28	44.93±3.07	18.38±2.39	31.67±2.70	9.24±0.24	44.16±3.61	11.89±1.19		
	0.14	103.69±5.35	96.43±1.89	105.31±2.59	83.74±1.54	110.73±7.60	85.92±6.39		
	0.07	101.45±4.59	101.27±1.51	108.73±4.05	99.18±0.81	105.58±4.20	91.42±7.32		
	0.04	105.38±1.62	104.81±2.92	104.32±3.57	102.29±2.71	99.05±8.70	100.62±5.04		
	0.02	99.91±1.64	101.38±3.85	102.16±1.80	98.30±3.64	97.01±2.92	92.56±7.38		
	0.48							41.75±1.54	34.69±1.87
	0.24							54.01±1.67	49.24±4.09
	0.12							84.12±3.89	76.92±2.50
	0.06							100.78±5.13	96.73±2.56
	0.03							103.06±3.90	102.05±4.08

Table S2. A spectroscopy-based structural interpretation in DPPC and DSPC liposomes after sterilization.

Lipid	Method	FTIR observations	Raman observations	Structural changes
DPPC	Autoclaving	Band positions preserved; intensities comparable; no new band; minor linewidth changes	Choline and CH envelope conserved	Conservative to molecular organization; at most slight compaction/reorientation
	UV	Similar to autoclaving; no new absorption; small FWHM changes	Largely preserved features	Minimal perturbation of chain order/headgroup environment
	Filtration	Attenuation at 721, ~1467, ~2849, ~2915–2916 cm^{-1}; weakening in 825–1162 cm^{-1}; modest CH_2 peak broadening	Choline band diminished; reduced at ~2950 cm^{-1}	Increased gauche disorder; partial headgroup/chain reorganization; strongest perturbation among methods

DSPC	Autoclaving	Positions/intensities preserved; no new bands	Preserved choline/CH features	Conservative effect; organization maintained
	UV	Similar to autoclaving	Preserved features	Minimal perturbation
	Filtration	Attenuation and CH_2 broadening more pronounced than DPPC; weakening at 825–1162 cm^{-1}	Stronger choline attenuation; reduced at ~2950 cm^{-1}	Marked chain-order disruption and headgroup reorganization; filtration impact > DPPC
